# Pan beriberi in a Young Male: A Case Report

**DOI:** 10.1002/ccr3.72033

**Published:** 2026-02-09

**Authors:** Sonam Yangzom, Dechen Wangmo, Diki Dolkar, Kezang Tshomo, Dechen Pelden, Tenzin Kelzang

**Affiliations:** ^1^ Department of Internal Medicine Eastern Regional Referral Hospital Mongar Bhutan; ^2^ Eastern Regional Referral Hospital Mongar Bhutan

**Keywords:** beriberi, Bhutan, pan beriberi, thiamine, vitamin B12

## Abstract

Pan beriberi should be considered in young patients with unexplained acute heart failure, neurological deficits, and gastrointestinal pseudo‐obstruction, particularly in malnutrition or chronic alcohol use. Early clinical recognition and prompt high dose thiamine therapy can be lifesaving, even where laboratory confirmation is unavailable.

## Graphical abstract

Chest X‐ray findings of cardiomegaly and pulmonary congestion in pan beriberi reflecting reversible cardiac involvement due to thiamine deficiency.

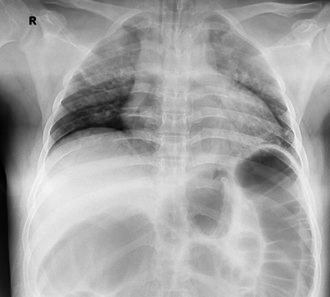

## 1 Introduction

Thiamine was the first identified vitamin that acts as a co‐enzyme in carbohydrate metabolism, helping in the generation of energy. It helps in the decarboxylation of amino acids and alpha‐keto acids [1]. It also plays an important role in the nervous system, where it helps in the synthesis of neurotransmitters [2].

Thiamine is present in many foods, including yeast, pork, legumes and brown rice. Thiamine has a short half‐life of 2–3 weeks and the daily requirement is 2 mg. Inadequate intake or excessive loss can lead to thiamine deficiency [3]. It can occur in people with a low vitamin diet, especially seen in the Asian population, where polished rice and ground grains are the main staple diet. At‐risk population includes people who are malnourished with chronic alcoholism, those who have undergone gastrointestinal surgeries and pregnant women, especially during the first trimester [4].

Thiamine deficiency can present with a wide spectrum of neurological, cardiovascular, and gastrointestinal manifestations. Neurologically, patients may develop confusion, ataxia, and.ophthalmoplegia [5], cardiovascular presentations such as high‐output heart failure, pulmonary edema [6] and gastrointestinal symptoms such as recurrent vomiting, abdominal discomfort, and functional ileus [7].

## 2 Case Report

A 26‐year‐old man with no known comorbidities presented with 3 days of severe dizziness, double vision, tinnitus, and confusion. He also reported progressive bilateral lower limb weakness and numbness for 2 months. He consumed alcohol regularly and reported a predominantly carbohydrate‐based diet with minimal protein and vegetable intake.

### 2.1 Initial Examination

On arrival, he was tachycardic (heart rate of 130/min), normotensive (blood pressure of 130/86 mm of mercury), afebrile, with an oxygen saturation of 94% on room air. Neurological examination revealed bilateral abducent and oculomotor nerve palsies, bilateral internuclear ophthalmoplegia, gait ataxia, and lower limb weakness (Muscle Power of 4/5, flaccid paraparesis with areflexia).

### 2.2 Cardiovascular Course

**FIGURE 1:**
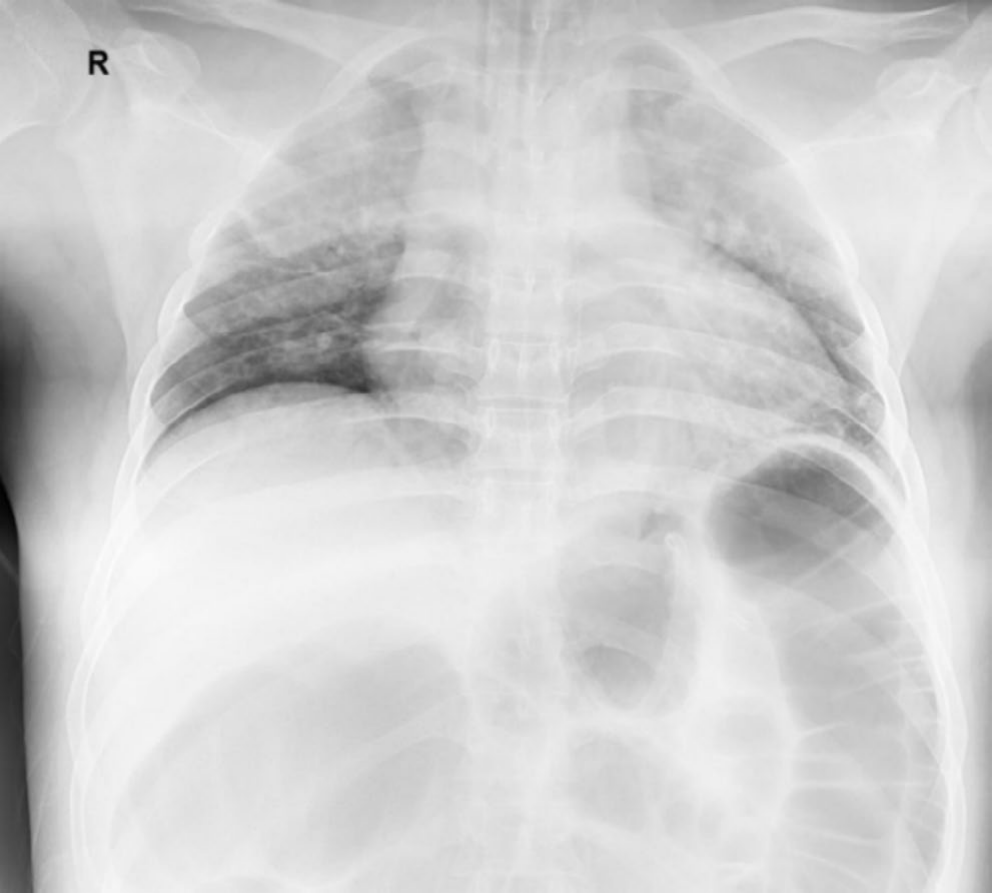
Chest X‐ray demonstrates cardiomegaly and pulmonary congestion.

Within days, he developed acute pulmonary oedema and type 1 respiratory failure, requiring high‐flow oxygen followed by intubation. Chest X‐ray demonstrated cardiomegaly and pulmonary congestion (Figure 1), while echocardiography showed global left ventricular hypokinesia, preserved ejection fraction (50%), grade II diastolic dysfunction, and left atrial dilatation. Arterial blood gas revealed hyperlactatemia (6.3 mmol/L) without features of Acute Respiratory Distress Syndrome (ARDS). He required intubation twice and was successfully extubated after supportive care.

A follow‐up transthoracic echocardiogram performed 12 days after clinical recovery showed resolution of the left atrial dilatation and diastolic dysfunction, with stable mildly reduced left ventricular systolic function (ejection fraction 49%) and no valvular dysfunction.

### 2.3 Neurological Manifestations

Despite a normal Computed Tomography of the brain and cerebrospinal fluid study, his ophthalmoplegia, intermittent confusion, and ataxia were consistent with Wernicke's encephalopathy.

### 2.4 Gastrointestinal Manifestations

**FIGURE 2:**
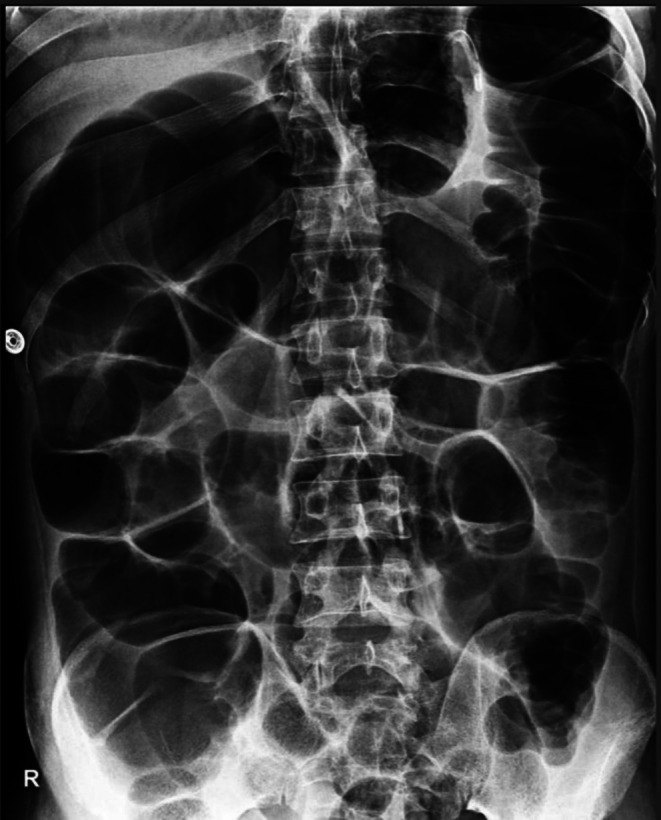
Dilated small and large bowel loops (6.0 cm in AP diameter) without obstructive lesions.

During his stay in the intensive care unit, he developed abdominal distension, nausea, and vomiting. Abdominal imaging revealed dilated small and large bowel loops (6.0 cm in anteroposterior (AP) diameter) without obstructive lesions, consistent with functional obstruction/pseudo‐obstruction (Figure 2). Acute abdomen, sepsis and hypokalemia‐related intestinal ileus were also considered, but imaging and laboratory results did not support these diagnoses.

### 2.5 Peripheral Neuropathy

The patient reported progressive lower limb weakness for months before presentation. Examination confirmed flaccid paraparesis with sensory symptoms, areflexia, and equivocal plantar responses, consistent with dry beriberi.

Alcohol‐related rhabdomyolysis was also considered but deemed unlikely due to the absence of myalgia, dark‐colored urine, renal deterioration that would be attributable to muscle injury, and the rapid neurological improvement following thiamine replacement.

### 2.6 Management

High‐dose intravenous (IV) thiamine was initiated at 200 mg four times daily when he had features of wet beriberi and features of Wernicke's encephalopathy. Later that day, it increased to 500 mg three times daily (total 1500 mg/day). The patient demonstrated rapid clinical improvement following thiamine replacement.

### 2.7 Timeline

**Table:** 

Time	Clinical event
2 months prior	Progressive bilateral lower limb weakness and numbness
3 days prior	Onset of dizziness, diplopia, tinnitus, and confusion
Day 0 (Admission)	Tachycardia, ophthalmoplegia, ataxia, flaccid paraparesis; CT brain and CSF normal
Days 1–2	Acute pulmonary oedema and type 1 respiratory failure; hyperlactatemia (6.3 mmol/L); intubation required
Day 2	Echocardiography: global LV hypokinesia with preserved EF; diagnosis of pan beriberi; IV thiamine initiated
Days 3	Rapid neurological improvement; resolution of confusion
Day 4	Complete resolution of ophthalmoplegia; successfully extubated
Day 5	Resolution of gastrointestinal symptoms
Day 7	Ambulating independently; marked clinical recovery
Day 14	Follow‐up transthoracic echocardiography demonstrated resolution of left atrial and ventricular dilatation and diastolic dysfunction

## 3 Discussion

This case illustrates the spectrum of multisystemic beriberi, encompassing wet, dry, Wernicke's, and gastrointestinal forms in a single patient. Wet beriberi results from impairment in the energy metabolism of the myocardium, leading to heart failure, pulmonary oedema, and lactic acidosis [6]. Wernicke's encephalopathy, characterized by the triad of ophthalmoplegia, confusion, and ataxia, is a neurological emergency requiring immediate administration of high‐dose IV thiamine [8, 9]. Gastrointestinal beriberi manifests as pseudo‐obstruction or paralytic ileus due to impaired energy metabolism in enteric neurons [7]. Dry beriberi causes symmetrical peripheral neuropathy with motor weakness, sensory loss, and areflexia [9].

Our patient's presentation was initially confusing due to overlapping features [8]. The key diagnostic clues were chronic alcohol consumption, hyperlactatemia, and rapid response to thiamine.

In a case series by Dabar et al. [10], four critically ill, non‐septic patients developed severe lactic acidosis and acute fulminant beriberi. Similarly, our patient presented with hyperlactatemia alongside multisystem involvement. These cases collectively emphasize the importance of considering thiamine deficiency in patients with unexplained lactic acidosis without obvious causes.

Unlike fulminant *Shoshin beriberi*, which causes catastrophic cardiovascular collapse [10], our patient developed subacute heart failure with preserved ejection fraction and prominent multisystem involvement. The resolution of chamber dilatation and diastolic dysfunction on follow‐up echocardiography supports a reversible metabolic cardiomyopathy rather than primary structural heart disease.

Importantly, early recognition and thiamine replacement reversed potentially fatal complications. In a study by Smithline et al. [11], patients with acute heart failure were administered intravenous thiamine, resulting in significant improvements in dyspnea and left ventricular ejection fraction. This mirrors our approach, where rapid thiamine administration led to marked clinical improvements.

## 4 Conclusion

This case highlights the varied manifestations of thiamine deficiency. Despite being in a resource‐limited setting without access to serum thiamine measurements, we were able to promptly diagnose pan beriberi based on clinical features and achieve rapid reversal with high‐dose intravenous thiamine. This case emphasizes the critical importance of early recognition and timely treatment, as delayed intervention can result in significant morbidity or mortality. Clinicians should maintain a high index of suspicion for thiamine deficiency in patients presenting with unexplained lactic acidosis, history of alcoholism, cardiac dysfunction, or neurological deficits, even when laboratory confirmation is not available.

## Author Contributions

**Sonam Yangzom:** conceptualization, data curation, investigation, supervision, writing – review and editing. **Dechen Wangmo:** formal analysis, writing – original draft, writing – review and editing. **Diki Dolkar:** formal analysis, writing – original draft, writing – review and editing. **Kezang Tshomo:** formal analysis, writing – original draft, writing – review and editing. **Tenzin Kelzang:** formal analysis, writing – original draft, writing – review and editing. **Dechen Pelden:** formal analysis, writing – original draft, writing – review and editing.

## Funding

The authors have nothing to report.

## Disclosure

A version of this manuscript has been published as a preprint on Authorea (https://doi.org/10.22541/au.176656346.67761216/v1).

## Consent

Written informed consent was obtained from the patient for publication of this case report and accompanying images.

## Conflicts of Interest

The authors declare no conflicts of interest.

## Data Availability

Data sharing not applicable to this article as no datasets were generated or analyzed during the current study.
